# Genetic diversity and population structure analyses of *Plectranthus edulis* (Vatke) Agnew collections from diverse agro-ecologies in Ethiopia using newly developed EST-SSRs marker system

**DOI:** 10.1186/s12863-018-0682-z

**Published:** 2018-10-11

**Authors:** Fekadu Gadissa, Kassahun Tesfaye, Kifle Dagne, Mulatu Geleta

**Affiliations:** 1 Department of Microbial, Cellular and Molecular Biology, Addis Ababa University, Box 1176, Addis Ababa, Ethiopia; 2 Department of Biology, Madda Walabu University, Box 247, Bale Robe, Ethiopia; 3 Ethiopian Biotechnology Institute, Ministry of Science and Technology, Box 32853, Addis Ababa, Ethiopia; 4 Department of Plant Breeding, Swedish University of Agricultural Sciences, Box 101, SE-23053 Alnarp, Sweden

**Keywords:** Ethiopian dinich, Expressed sequence tags, Genetic diversity, *Plectranthus edulis*, Population structure, Simple sequence repeats

## Abstract

**Background:**

*Plectranthus edulis* (Vatke) Agnew (locally known as Ethiopian dinich or Ethiopian potato) is one of the most economically important edible tuber crops indigenous to Ethiopia. Evaluating the extent of genetic diversity within and among populations is one of the first and most important steps in breeding and conservation measures. Hence, this study was aimed at evaluating the genetic diversity and population structure of this crop using collections from diverse agro-ecologies in Ethiopia.

**Results:**

Twenty polymorphic expressed sequence tag based simple sequence repeat (EST-SSRs) markers were developed for *P. edulis* based on EST sequences of *P. barbatus* deposited in the GenBank. These markers were used for genetic diversity analyses of 287 individual plants representing 12 populations, and a total of 128 alleles were identified across the entire loci and populations. Different parameters were used to estimate the genetic diversity within populations; and gene diversity index (GD) ranged from 0.31 to 0.39 with overall mean of 0.35. Hierarchical analysis of molecular variance (AMOVA) showed significant but low population differentiation with only 3% of the total variation accounted for variation among populations. Likewise, cluster and STRUCTURE analyses did not group the populations into sharply distinct clusters, which could be attributed to historical and contemporary gene flow and the reproductive biology of the crop.

**Conclusions:**

These newly developed EST-SSR markers are highly polymorphic within *P. edulis* and hence are valuable genetic tools that can be used to evaluate the extent of genetic diversity and population structure of not only *P. edulis* but also various other species within the Lamiaceae family. Among the 12 populations studied, populations collected from Wenbera, Awi and Wolaita showed a higher genetic diversity as compared to other populations, and hence these areas can be considered as hot spots for in-situ conservation as well as for identification of genotypes that can be used in breeding programs.

**Electronic supplementary material:**

The online version of this article (10.1186/s12863-018-0682-z) contains supplementary material, which is available to authorized users.

## Background

Ethiopia is one of the countries in the world that has the greatest crop genetic diversity and considered as a primary gene center for several crops [1–3] including edible roots and tubers [4]. However, the food potential of horticultural crops, particularly those of indigenous edible roots and tubers have not been fully exploited despite their significant contributions to the livelihood of subsistence farmers. Such crops are overlooked in terms of research and breeding and their production and management systems are restricted to local farmers’ varieties maintained by farmers using local knowledge [5–7].

*Plectranthus edulis* (Vatke) Agnew (Lamiaceae), also locally known as Ethiopian potato *syno*. Ethiopian dinich, is one of the most economically important indigenous tuber crops [2, 8] with wide distribution in parts of Africa, largely in wild form [9]. It produces edible stem tubers on stolon below ground. The crop can thrive and set tubers under significant environmental constraints including degraded and poor soil and can produce reasonable yield without a need for intensive management practices [10].

In Ethiopia, this edible tuber crop is commonly cultivated by smallholder farmers around homesteads, alone or mixed with cereals, fruits and pulses, largely for household consumption and rarely for marketing of its tubers. It was one of the most common Ethiopian staple crops [11] and usually called ‘hunger crop’ as it fills the food supply gap that occurs from August to November, the period before the harvest of cereal crops. It has also been widely used as a folk medicine in several parts of Africa including Ethiopia [12] and is commonly visited by honeybees for its nectar [13].

Nowadays, local gene pool of the crop is under the threat of severe genetic erosion as it is disappearing from several areas where it used to be widely cultivated and restricted to highly marginalized land and limited to few elderly farmers in some other areas. The lack of improved planting material and limited awareness about the significance of this crop among younger farmers as well as the introduction of other high yielding tuber crops like Irish potato to the area, recurrent drought and environmental degradation have contributed to the genetic erosion of this crop [5, 14, 15].

Assessing the extent of genetic diversity of crop species is a vitally important step for their effective conservation and improvement. Molecular markers, such as simple sequence repeats (SSRs) are among the most important genetic tools for such purpose [16, 17]. SSRs derived from Expressed sequence tags (ESTs) (EST-SSRs) have been widely used in many plant species including root and tuber crops, as they are relatively fast and cost-effective to develop [18], have a well-conserved flanking sequences among phylogenetically closely related species and hence are highly transferable among related taxa, and are less susceptible to null alleles [19]. Such cross-genera and cross-species transferable molecular markers are highly important genomic resources to study plant species with little or no DNA sequence information, such as *P. edulis*. Until recently, very few transferable EST-SSR markers have been developed within the family Lamiaceae [20, 21] and, so far, there is no report of such markers for the genus *Plectranthus.* Moreover, there is no publication on molecular marker based genetic diversity of *P. edulis*, which shows that researchers and plant breeders have not given enough attention for conservation and improvement of this crop. Consequently, its production, utilization, and improvement are highly restricted. Hence, this study was initiated with the aim of developing and validating EST-SSR markers for use in population structure and genetic diversity analyses of the crop, which eventually serve as a basis for its improvement and sustainable conservation.

## Methods

### Plant material

**Fig. 1:**
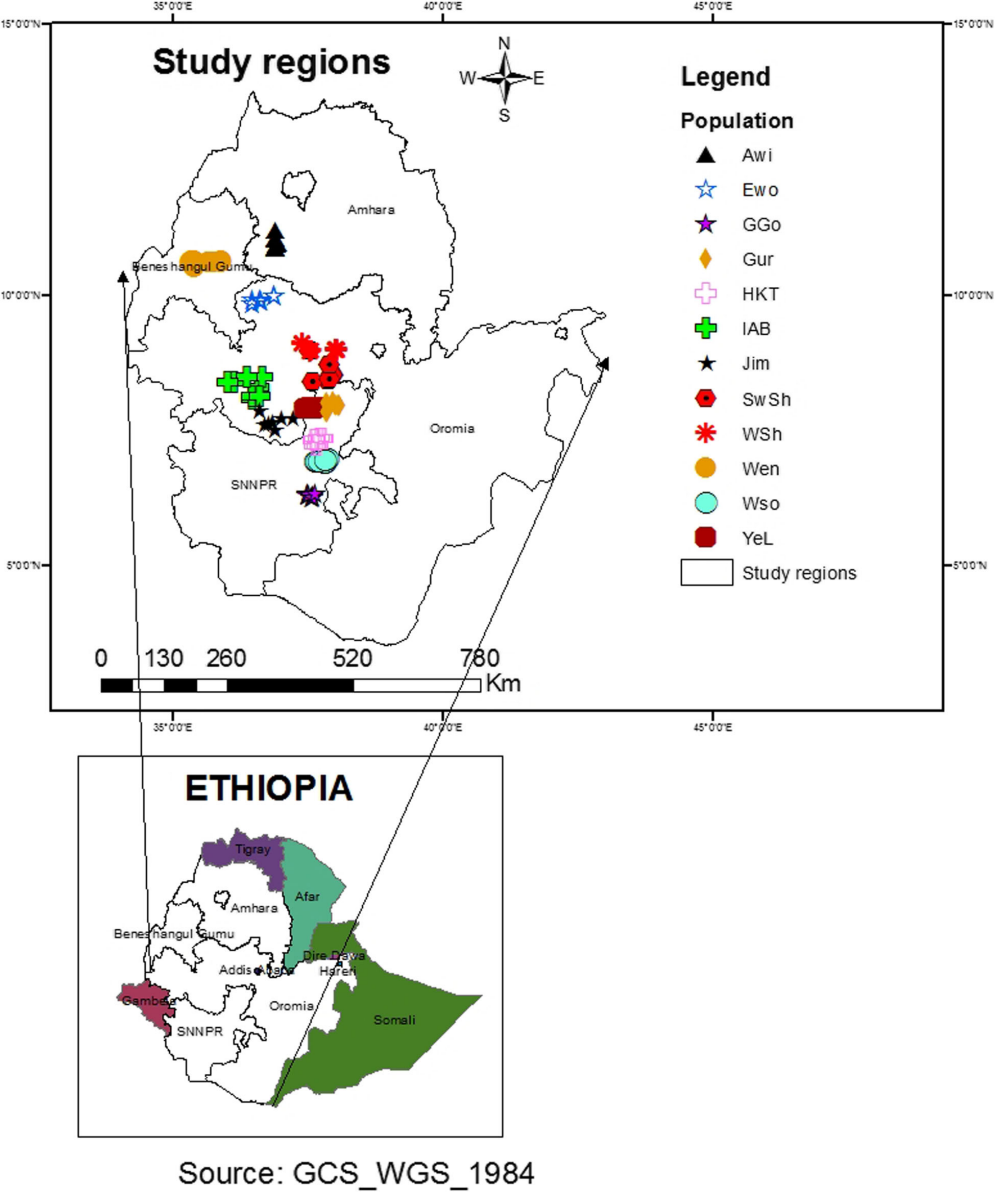
Map of Ethiopia showing its Federal Regions (left bottom) and tuber sample collection sites, representing the 12 studied populations, within four of the Federal Regions (left upper) (see Table 1 for full description of the populations). The map was original and constructed using geographic coordinates and elevation data gathered from each collection sites using global positioning system (GPS)

A total of 174 tuber samples of cultivated *P. edulis*, representing 12 populations, were randomly collected from farmers’ fields with permission from individual farmers (Fig. 1, Table 1). The identity of the samples was confirmed based on the species description provided in Flora of Ethiopia and Eritrea [22]. The collected samples were planted during the regular crop growing season (end of April) in 2016 at Holeta Agricultural Research Centre (which is a part of the Ethiopian Institute of Agricultural Research) field site located 40 Km west of Addis Ababa. This field site is located at a geographic position of 09^o^04’N, 38^o^ 29′E and altitude of 2400 masl.

**Table 1:** List of populations used in this study and their geographic positions and altitudes of collection along with their tuber and leaf sample sizes

Population	Sample size		Altitude range (m)	Geographic position (UTM)^a^	
	Tuber	Leaf		Latitude range	Longitude range
*SwSh*	13	24	1817–2631	0373275–0386714	0927865–0962106
*EW*	14	24	1991–2172	0221199–0266764	1,089,267–1,105,086
*Aw*	21	25	2167–2643	0267210–0281621	1,201,272–1,239,531
*Gur*	18	24	2069–2929	0371621–0397588	0867217–0889992
*HKT*	9	23	2121–2562	0363184–0370744	0813134–0832296
*IAB*	14	25	1650–2096	0133324–0784937	0893136–0949107
*Jim*	16	24	1605–2228	0197291–0327491	0829838–0875685
*GG*	13	24	2513–2744	0332363–0343464	0690582–0697778
*WS*	18	23	1979–2232	0349195–0372876	0760650–0770184
*Wen*	10	26	2476–2530	0789433–0793823	1,174,041–1,175,300
*WSh*	17	23	2249–2924	0322544–0394336	0989176–1,007,305
*YeL*	11	22	2466–2589	0337470–0339720	0873081–0875666
Total	174	287			

*SwSh* Southwest Shewa, *EW* Esat Wollega, *Aw* Awi, *Gur* Gurage, *HKT* Hadiya, Kembata and Tembaro, *IAB* Illu Aba Bora, *Jim* Jimma, *GG* Gamo Gofa, *WS* Wollaita Sodo, *Wen* Wenbera, *WSh* West Shewa, *YeL* Yem Liyu; ^a^UTM = Universal Transverse Mercator coordinate system

### Leaf sample collection and DNA extraction

oung leaf tissue from 287 individual plants (one to three individual plants per tuber sample) (Table 1, Additional file 1) were separately collected in a ziplock bag, silica gel dried and transported to the Swedish University of Agricultural Sciences (SLU), Alnarp, Sweden. Genomic DNA was extracted from these samples using a modified Cetyl Trimethyl Ammonium Bromide (CTAB) protocol as described in Geleta et al. [23]. The DNA quality was assessed using 1% agarose gel electrophoresis whereas NanoDrop® ND-1000 Spectrophotometer (Saveen Warner, Sweden) was used to determine the quantity of extracted DNA.

### Data mining, designing and screening EST-SSR primers

Initially, 3263 *P. barbatus* EST sequences were retrieved from the National Centre for Biotechnology Information (NCBI public DNA sequence database (https://www.ncbi.nlm.nih.gov/nucest/?term=Plectranthus+barbatus), and sequences containing SSRs were screened using WebSat, a web-based software for microsatellite marker development [24] (http://purl.oclc.org/NET/websat/). After excluding redundant, overlapping and short sequences, about 300 sequences containing two to six SSR motifs were identified. Of these, 40 sequences were selected for designing primers using Primer3 primer designing program [25, 26] (http://bioinfo.ut.ee/primer3-0.4.0/).

The 40 newly designed primer-pairs were tested for amplification of their target genomic regions using 36 DNA samples representing the 12 populations of *P. edulis*. The polymerase chain reaction (PCR) products were electrophoresed on 1.5% agarose gel containing gel-red and visualized using Saveen Werner AB UV camera equipped with SSM930CE Sony Black and white Monitor. The size of amplified products was estimated by loading GeneRuler 50 bp DNA ladder together with the samples on separate lanes. Under optimized PCR conditions, 20 of the 40 primer-pairs consistently amplified their polymorphic loci, and hence were selected for use on all samples included in the present study (Table 2).

**Table 2:** Characteristics of the 20 polymorphic SSR markers developed for genetic diversity analyses of *P. edulis*

Primer / locus name	GenBank ANSS^a^	SSR motif (5′ to 3′)	forward primer (5′ to 3′)	reverse primer (5′ to 3′)	Expected allele size (bp)	Observed allele size range (bp)	T_a_ (°C)
PE_01	GB|JZ730850.1	(TC)9	TCACCGCAGTTTTCAGTCTCTA	ATGAGATTCGCCATAGGTTTGT	102	122–146	59
PE_02	GB|JZ730431.1	(AAG)6	TGAATCTGCAAAGACATCTGCT	AACTGAGACAACTCCATTGACG	114	108–126	57
PE_03	GB|JZ730426.1	(TA)10	GAGACGACGACCAATGTTGTTA	CTCTCTATCACTCCTGACGGCT	125	106–142	56
PE_04	GB|JZ732709.1	(ACACCC)5	GGGGAATTAGAGATGGAAAGATAGA	TTGAGGGTGTGAAGTGGTACAG	131	137–143	57
PE_05	GB|JZ732789.1	(TG)10	GCCGTATCTCCATTTGTTGATT	TCCATGCTCCTCACACATTATC	141	140–168	57
PE_06	GB|JZ731502.1	(AC)6	GCTTACGCCAAGAACTGAAACT	AATAGCAATCTCTTTCCCTCCC	164	162–182	55
PE_07	GB|JZ730947.1	(CTC)4	GTTGGACGACTGGGTTTTATGT	GAATGACGCTAGTTTGCTGTTG	314	319–340	55
PE_08	GB|JZ731328.1	(GCT)6	CCAACAGCAATCCATATTACCA	ATTTCTCAAGTCAGTCCGAGGT	172	162–189	57
PE_09	GB|JZ732633.1	(CAG)6	AACCAAATGACAGGAGCATCTT	CAATTTCTTCATACTGGGTGGC	184	180–206	55
PE_10	GB|JZ730151.1	(CTT)5	GGGTAACGATTTGAATAATGCG	GTGAAGCGGGATCTACACTGA	188	176–200	52
PE_11	GB|JZ730075.1	(AGAGA)4	GTCAGCCTTTCTCTCTCGTCTC	AGGGGAGTGTGTTATCAAATGG	223	249–264	56
PE_12	GB|JZ730223.1	(AGC)6	GACCATCGGTAAGGAGAACTTG	GGATATGAGCTGGATAGCTGGT	226	213–243	55
PE_13	GB|JZ731331.1	(CAC)5	GCAAAAGTTCTACCAGCGTTTC	GGTTTGTTGATCCCAACGTAAT	229	242–251	57
PE_14	GB|JZ731120.1	(GAA)10	AATTACTTTCATGTCCAACGCC	TTTTCATCACTACCATCCCAGTC	229	228–243	55
PE_15	GB|JZ731840.1	(TGT)5	AGTTGAGATTGTACTCCACGTTGT	CTACTTCAGTTCCGGCCTCTTA	260	233–260	57
PE_16	GB|JZ732555.1	(AC)9	GGCGATATTCAAGAAAGCAACT	TCTTTTCACGCTTCCATCTCTT	355	319–339	57
PE_17	GB|JZ729809.1	(GA)8	GGGGCTTTTCTTGTTTGAAGAT	ATTGGAGGCAACTCATCAGAAT	361	370–382	57
PE_18	GB|JZ732589.1	(GA)9	ATGAAGAGTGAAGAGGCTGGAG	AGGAGCAAGCAAATAGAAATCG	306	304–322	57
PE_19	GB|JZ732378.1	(TGC)5	GTGTACATGCCATAGAATGAGT	GACTTCATCATATACGGCGGTT	167	180–192	57
PE_20	GB|JZ730154.1	(TGC)5	TACTTTATGGCAGAGAATGCGA	CTTGAGAGCCCTGAGACTTCAT	178	200–227	57

^a^*ANSS* Accession number of source sequences

### Pre-amplification and capillary electrophoresis

For cost effectiveness and improved quality of amplified products, the sequences of the 20 primer-pairs were modified as follows: (1) A 18-bp universal M13 primer sequence (5′-TGTAAAACGACGGCCAGT-3′) was added as a common tail to the 5′-end of all forward primers following Oetting et al. [27]; and (2) a PIG-tail sequence of 5′-GCTTCT-3′ was added to the 5′-end of the reverse primers to prevent non-templated addition of nucleotides to amplified products as described in Brownstein et al. [28]. M13 primer 5′-end labeled with HEX™ or 6-FAM™ fluorophores was used as a third primer in each PCR amplification.

PCR was carried out using 96-well plates with 25 μl reaction volume [1× reaction buffer, 1.5 mM MgCl_2_, 0.3 mM dNTPs, 0.08 μM M13-tailed forward primer, 0.3 μM pig-tailed reverse primer, 0.3 μM 6-FAM or HEX labeled M13 primer, 1 U Dream Taq DNA Polymerase, and 25 ng template DNA]. A mixture of all the components except genomic DNA was included as a negative control. Amplification was performed using GeneAMP PCR 9700 thermocycler (Applied Biosystems Inc. USA) according to the following five-stage PCR protocol: (1) initial 15 min denaturation at 95 °C, (2) 35 cycles of 30 s denaturation at 94 °C, 30 s annealing at optimized annealing temperature for each primer-pair (see Table 1), and 30 s primer extension at 72 °C, (3) eight cycles of 30 s denaturation at 94 °C, 45 s annealing at 53 °C and 45 s primer extension at 72 °C, (4) additional primer extension at 72 °C for 10 min, and (5) a final 30 min primer extension at 60 °C. The PCR products were stored at 4 °C until they were electrophoresed. The PCR products were multiplexed into panels based on fragment sizes of the SSRs and the fluorescence label of the M13 primer and diluted 25× using Millipore water. Finally, 0.7 μl of multiplexed and diluted PCR products, 1.9 μl Hi-Di formamide and 0.3 μl size standard (GenScanTm600 LIZ® size standard) were mixed and ccapillary electrophoresis was conducted using Genetic Analyzer 3500 (Applied Biosystems) at SLU, Department of Plant Breeding, Alnarp, Sweden.

### Allele scoring and statistical analysis

Peak identification and fragment sizing were done using GeneMarker V2.6.0 (SoftGenetics®) with default settings and 200 threshold intensities. Then, the allele size (bp) data at each locus were exported to excel for statistical analyses. Locus based diversity indices: major allele frequency (MAF), the number of alleles (NA), gene diversity (GD), and polymorphic information content (PIC) were determined using PowerMarker ver. 3.25 [29] (Table 3). The number of effective alleles (Ne), observed heterozygosity (Ho), expected heterozygosity (He) [30], Shannon’s Information Index (I), and estimate of the deviation from Hardy-Weinberg equilibrium (HWE) over the entire populations and population genetic diversity indices: Ne, percentage of polymorphic loci (PPL), Ho, He, I, Nei’s gene diversity over the entire loci were computed using GenAlEx ver. 6.501 [31] (Table 4). To determine the correlation between observed allelic diversity and sample size of populations, rarified allelic richness (Ar) and private rarified allelic richness (Ap) were estimated using HP-Rare 1.1 software [32]. To identify genetic groups, G, the number of distinct multi-locus genotypes (MLGs) present in each sample were evaluated using GenClone 2.0 [33]. To evaluate the global clonality rate of the sample, the index of clonal diversity was computed as G/N, where G is the number of MLGs and N is the total number of genotyped individuals. To analyze the distribution of genetic variation and to estimate the variance components of the populations, analysis of molecular variance (AMOVA) was computed using Arlequin ver. 3.5.2.2 [34]. Population differentiation tests: Wrights fixation index (F_ST_) and pairwise F_ST_ were computed using GenAlEx ver. 6.501 [31], and significance was tested based on 1000 bootstraps.

**Table 3:** Informativeness and levels of different diversity indices of the SSR loci across populations

SSR loci	MAF	NA	Ne	GD	Ar	Arp	Ho	He	F_ST_	Nm	PIC	I	P_HWE_^a^	F
PE_01	0.87	7	1.35	0.24	2.91	0.15	0.22	0.23	0.08	2.90	0.23	0.45	0.530^ns^	0.11
PE_02	0.49	6	2.24	0.55	3.05	0.13	0.97	0.55	0.01	35.07	0.45	0.87	0.000*	−0.75
PE_03	0.87	8	1.34	0.25	3.32	0.06	0.19	0.23	0.04	5.47	0.24	0.49	0.000*	0.23
PE_04	0.91	2	1.19	0.16	2.02	0.07	0.18	0.16	0.06	4.07	0.15	0.28	0.112^ns^	−0.07
PE_05	0.79	10	1.56	0.36	3.56	0.18	0.27	0.35	0.04	5.52	0.34	0.66	0.000*	0.27
PE_06	0.46	9	3.17	0.70	4.97	0.08	0.86	0.68	0.03	8.54	0.66	1.32	0.000*	−0.22
PE_07	0.97	9	1.06	0.05	1.72	0.46	0.01	0.05	0.04	5.67	0.05	0.12	0.783^ns^	0.73
PE_08	0.96	5	1.09	0.08	1.68	0.20	0.07	0.08	0.05	4.66	0.08	0.15	0.115^ns^	0.18
PE_09	0.68	6	2.04	0.50	4.28	0.08	0.38	0.49	0.03	7.47	0.47	0.95	0.000*	0.25
PE_10	0.72	5	1.70	0.42	2.43	0.11	0.54	0.40	0.05	5.23	0.34	0.62	0.000*	−0.29
PE_11	0.77	6	1.66	0.38	3.11	0.16	0.39	0.37	0.03	7.64	0.35	0.68	0.335^ns^	−0.01
PE_12	0.76	7	1.65	0.39	3.40	0.17	0.29	0.37	0.05	5.22	0.36	0.70	0.000*	0.26
PE_13	0.96	4	1.09	0.08	2.00	0.09	0.04	0.08	0.02	11.76	0.07	0.17	0.542^ns^	0.53
PE_14	0.81	4	1.44	0.30	2.24	0.07	0.30	0.28	0.09	2.69	0.26	0.46	0.965^ns^	0.04
PE_15	0.90	5	1.24	0.19	2.66	0.08	0.13	0.18	0.05	4.56	0.18	0.36	0.876^ns^	0.30
PE_16	0.70	12	1.80	0.46	4.03	0.30	0.49	0.43	0.06	3.70	0.42	0.81	0.115^ns^	−0.06
PE_17	0.63	6	1.99	0.51	2.90	0.06	0.57	0.48	0.06	3.87	0.44	0.78	0.000*	− 0.10
PE_18	0.68	8	1.96	0.49	3.40	0.26	0.43	0.48	0.03	8.65	0.44	0.85	0.128^ns^	0.11
PE_19	0.53	5	2.12	0.53	2.87	0.07	0.88	0.53	0.01	25.01	0.43	0.82	0.000*	−0.65
PE_20	0.72	4	1.65	0.41	2.22	0.07	0.54	0.38	0.06	4.11	0.33	0.59	0.000*	−0.33
Mean	0.76	6.45	1.67	0.35	2.94	0.14	0.39	0.34	0.04	5.84	0.32	0.61		−0.09

*MAF* Major allele frequency, *NA* Number of alleles, *Ne* Effective number of alleles, *GD* Gene diversity, *Ar* Allelic richness, *Arp* Private allelic richness, *H*_*O*_ Observed heterozygosity, *H*_*e*_ Expected heterozygosity, *Fst* Inbreeding coefficient within subpopulations relative to total (genetic differentiation among subpopulations), *Nm* Gene flow estimated from *Fst* 0.25(1- Fst)/Fst, *PIC* Polymorphic information content, *I* Shannon’s Information Index, *F* Fixation Index, *P*_*HWE*_^*a*^ *P*-value for deviation from Hardy Weinberg equilibrium, *ns* not significant, * = *P* < 0.0001 and hence highly significant

**Table 4:** Summary of different population diversity indices averaged over the 20 loci

Pop^a^	G	N	G/N	Ar	Arp	Ne	PPL	Ho	He	GD	I
*SwSh*	8	24	0.33	2.78	0.12	1.60	95.00	0.43	0.33	0.34	0.59
*EW*	9	24	0.38	2.56	0.01	1.60	90.00	0.37	0.30	0.31	0.52
*Aw*	13	25	0.52	2.93	0.14	1.80	95.00	0.35	0.36	0.36	0.64
*Gur*	10	24	0.42	3.33	0.28	1.60	95.00	0.33	0.35	0.35	0.64
*HKT*	11	23	0.48	2.80	0.04	1.70	95.00	0.43	0.34	0.35	0.60
*IAB*	16	25	0.64	2.80	0.09	1.70	90.00	0.39	0.34	0.34	0.59
*Jim*	13	24	0.54	3.18	0.25	1.60	95.00	0.39	0.34	0.35	0.62
*GG*	19	24	0.79	2.99	0.17	1.60	95.00	0.39	0.34	0.35	0.61
*WS*	15	23	0.65	3.17	0.14	1.80	95.00	0.41	0.37	0.37	0.66
*Wen*	14	26	0.54	2.74	0.13	1.80	95.00	0.42	0.38	0.39	0.65
*WSh*	12	23	0.52	3.01	0.22	1.60	90.00	0.37	0.31	0.32	0.57
*YeL*	17	22	0.77	2.96	0.15	1.60	100.00	0.37	0.32	0.33	0.57
Mean	13.08	287^c^	0.55	2.94	0.15	1.70	94.20	0.39	0.34	0.35	0.61

^a^*SwSh* Southwest Shewa, *EW* East Wollega, *Aw* Awi, *Gur* Gurage, *HKT* Hadiya, Kembata and Tembaro, *IAB* Illu Aba Bora, *Jim* Jimma, *GG* Gamo Gofa, *WS* Wollaita Sodo, *Wen* Wenbera, *WSh* West Shewa, *YeL* Yem Liyu woreda, *G* number of distinct multi-locus genotype (MLG), *N* number of samples genotyped, *G/N* the ratio of G to N, *c* total number of samples, *Ar* Allelic richness, *Arp* Private allelic richness, *Ne* effective number of alleles, *PPL* percentage of polymorphic loci, *H*_*O*_ observed heterozygosity, *H*_*e*_ expected heterozygosity, *GD* gene diversity, *I* Shannon diversity index

Simple matching dissimilarity coefficient-based Neighbor-Joining tree and Nei’s standard genetic distance (D_ST,_ corrected) [35] based Unweighted Pair Group Method with Arithmetic Mean (UPGMA) [36] tree was constructed using DARwin var. 6.0.14 [37] and POPTREE2 [38], respectively, and significance was tested based on 1000 bootstraps [39]. The resulting trees were displayed using TreeView (Win 32) 1.6.6 program [40] and FigTree var. 1.4.3 [41]. Gene flow (Nm) among populations was estimated using the formula, Nm = 0.25(1 − Fst)/Fst [42].

A Bayesian model-based clustering algorithm in STRUCTURE ver. 2.3.4 [43, 44] was applied to infer the pattern of population structure and detection of admixture. To determine the most likely number of populations (K), a burn-in period of 50,000 was used in each run, and data were collected over 500,000 Markov Chain Monte Carlo (MCMC) replications for K = 1 to K = 12 using 20 iterations for each K. The optimum K value was predicted following the simulation method of Evanno et al. [45] using the web-based STRUCTURE HARVESTER ver. 0.6.92 [46]. Bar plot for the optimum K was determined using Clumpak beta version [47].

## Results

### Validation of the EST-SSRs marker and evaluation of their levels of polymorphism

The 20 newly developed EST-SSRs markers (Table 2) are predominantly trinucleotides and dinucleotides. Trinucleotide SSRs accounted for 55% of the loci with the number of repeats ranging from four to ten whereas dinucleotide SSRs accounted for 35% of the loci with the number of repeats ranging from six to ten. The remaining two loci (10%) were pentanucleotide and hexanucleotide repeats with four and five number of repeats, in that order. All the 20 loci were polymorphic and produced a total of 128 alleles (an average of 6.4 alleles per locus) (Table 3), out of which 62 (48.4%) were rare (frequency < 0.01) and 22 (17.2%) were scarce (frequency between 0.01 and 0.05). The frequency of seven alleles (5.5%) was between 0.05 and 0.1 whereas 37 alleles (28.9%) had a frequency of 0.1 or higher (Additional file 2).

The maximum number of alleles detected per locus was 12 (PE_16; Table 3). The least major allele frequency (MAF; 0.46), and largest effective number of alleles (Ne) (3.17), allelic richness (4.97), Nei’s gene diversity (GD) (0.70), polymorphic information contents (PIC) (0.66) and Shannon information index (I) (1.33) were recorded for PE_06. The highest MAF (0.97), private allelic richness (0.46), and the least Ne (1.06), I (0.12), GD (0.05) and PIC (0.05) were recorded for PE_07. In terms of the overall PIC, one SSR locus (PE_06) was found to be highly informative (PIC ≥0.5), 12 loci (60%) were moderately informative (0.5 < PIC ≥0.25), and the remaining seven were less informative (PIC < 0.25) (Table 3). The highest observed heterozygosity (Ho) (0.97), the lowest fixation index (− 0.75) and the highest value for gene flow (Nm) (35.1) were recorded for PE_02 (Table 3). Ten of the twenty loci showed a highly significant deviation from HW-equilibrium over the entire populations (Table 3).

### Genetic variation within and among populations

Among the 12 populations studied, not much differences were observed in terms of a number of genetic diversity paramters, such as effective number of alleles (Ne), observed heterozygosity (Ho) and expected heterozygosity (He), gene diversity (GD) and Shannon diversity index (I). However, *Wen*, *WS* and *Aw* populations scored higher values in Ne, GD and I while *SwSh* and *HKT* populations scored a higher Ho as compared to the other populations. Five populations: *Aw*, *Gur*, *EW*, *WSh*, and *YeL*, in the order of magnitude, scored slightly less than the mean Ho whereas *IAB*, *Jim*, and *GG* populations had a mean Ho value. Although Ho value is the lowest for *Gur* population, it comes first in terms of allelic richness (Ar) including richness in private alleles (Arp) with Ar of 3.33 and Arp of 0.28 (Table 4). *Jim* and *WSh* populations ranked second and third in terms of overall Arp. The mean number of distinct multi-locus genotypes (MLGs) and index of clonality (G/N) over the entire samples (populations) was 13.08 and 0.55, respectively (Table 4). The analysis of percentage of polymorphic loci (PPL) showed that at least 90% of the loci were polymorphic in each population studied, with a mean PPL of 94.2% (Table 4).

### Population genetic differentiation and gene flow

The hierarchical AMOVA was conducted without grouping the populations as well as by grouping the populations according to administrative regions (AR) and geographical regions (GR). In all cases, variation within individuals accounted for at least 97% of the total variation. The variation among populations, AR and GR accounted for only 3, 2 and 2% of the total variation, respectively. Hence, most of the within population variation is due to the heterozygosity of the individuals within each population. The overall F_ST_ value was very small (0.03) (Table 5, Additional file 3). The overall gene flow among the populations was estimated to be high (Nm = 5.84) (Table 3) whereas pairwise population differentiation test computed according to Weir [30], with exclusion of null alleles, ranged from 0.01 to 0.07.

**Table 5:** Analysis of molecular variance (AMOVA) for the 12 populations without grouping and with grouping them according to their geographic regions of collections (GR) and administrative regions of collections (AR) based on data from the 20 loci

Source of Variation	Df	SS	Variance components	%Variation	Fixation index	*P* value
Among populations	11	61.94	0.072 Va	2.55	F_ST_: 0.03	Va and F_ST_ = 0.000
Among individuals within populations	275	603.72	−0.56 Vb	−19.66	F_IS_: −0.20	Vb and F_IS_ = 1.000
Within individuals	287	948.50	3.31 Vc	117.11	F_IT_: −0.17	Vc and F_IT_ = 1.000
Total	573	1614.17	2.82			
Among AR	3	26.19	0.05 Va	1.85	F_ST_: 0.02	Va and F_ST_ = 0.000
Among individuals within AR	283	639.48	−0.52 Vb	−18.44	F_IS_: −0.19	Vb and F_IS_ = 1.000
Within individuals	287	948.50	3.31 Vc	116.59	F_IT_: −0.17	Vc and F_IT_ = 1.000
Total	573	1614.17	2.84			
Among GR	3	24.42	0.04 Va	1.53	F_ST_: 0.02	Va and F_ST_ = 0.000
Among individuals within GR	283	641.25	0.52 Vb	−18.37	F_IS_: −0.19	Vb and _FIS_ = 1.000
Within individuals	287	948.50	3.31 Vc	116.84	F_IT_: −0.17	Vc and F_IT_ = 1.000
Total	573	1614.17	2.83			

*AR* administrative regions (Oromia, Amhara, SNNPs, and Benshangul Gumuz), *GR* geographic regions (Northwestern, Western, Southern, and Southwestern Ethiopia), *Df* Degrees of freedom, *SS* Sum of squares

### Genetic distance between the populations

Nei’s standard genetic distance between populations ranged from 0.02 to 0.05. The highest pairwise genetic distance (0.05) was observed between four pairs of populations (*Aw* vs *WS*, *Wen* vs *HKT*, *WS* and *YeL*). The mean genetic distance of each population from the other populations ranged from 0.02 to 0.04 and, in terms of this parameter, *Wen* is the most distantly related population with a mean genetic distance of 0.04 (Table 6). Similarly, Weir’s estimation of population differentiation (F_ST_) [30, 48] ranged from 0.01 to 0.07 with the highest value recorded between *Wen* and *YeL* populations.

**Table 6:** Nei’s standard genetic distance between populations (below diagonal) [35], and Weir [30] estimation of population pairwise F_ST_ using the ENA correction as described in Chapuis and Estoup [48] (above diagonal) for the 12 populations studied

Pop ID	*SwSh*	*EW*	*Aw*	*Gur*	*HKT*	*IAB*	*Jim*	*GG*	*WS*	*Wen*	*WSh*	*YeL*	Mean^a^
*SwSh*	****	0.02	0.04	0.03	0.01	0.01	0.02	0.04	0.03	0.05	0.02	0.03	0.02
*EW*	0.01	****	0.04	0.02	0.01	0.01	0.02	0.03	0.03	0.05	0.01	0.02	0.02
*Aw*	0.03	0.03	****	0.04	0.04	0.03	0.03	0.04	0.06	0.02	0.03	0.05	0.03
*Gur*	0.02	0.01	0.03	****	0.03	0.03	0.02	0.02	0.01	0.05	0.01	0.01	0.02
*HKT*	0.01	0.01	0.03	0.02	****	0.01	0.02	0.05	0.01	0.06	0.01	0.03	0.02
*IAB*	0.01	0.01	0.02	0.02	0.01	****	0.01	0.04	0.03	0.05	0.02	0.02	0.02
*Jim*	0.02	0.02	0.02	0.02	0.02	0.01	****	0.03	0.03	0.04	0.01	0.02	0.02
*GG*	0.03	0.02	0.03	0.02	0.03	0.03	0.03	****	0.04	0.04	0.01	0.04	0.03
*WS*	0.03	0.02	0.05	0.02	0.02	0.03	0.03	0.03	****	0.06	0.02	0.03	0.03
*Wen*	0.04	0.04	0.02	0.04	0.05	0.04	0.04	0.04	0.05	****	0.04	0.07	0.04
*WSh*	0.02	0.01	0.02	0.01	0.02	0.02	0.01	0.01	0.02	0.03	****	0.02	0.02
*YeL*	0.02	0.02	0.03	0.01	0.03	0.02	0.02	0.03	0.03	0.05	0.02	****	0.03

All pairwise F_ST_ and genetic distance values are significant at *P* = 0.05

*ENA* Exclusion of null alleles

^a^ Mean Nei’s standard genetic distance of each population from the other populations; **** = not applicable

### Cluster analysis, PCoA, and population genetic structure

**Fig. 2:**
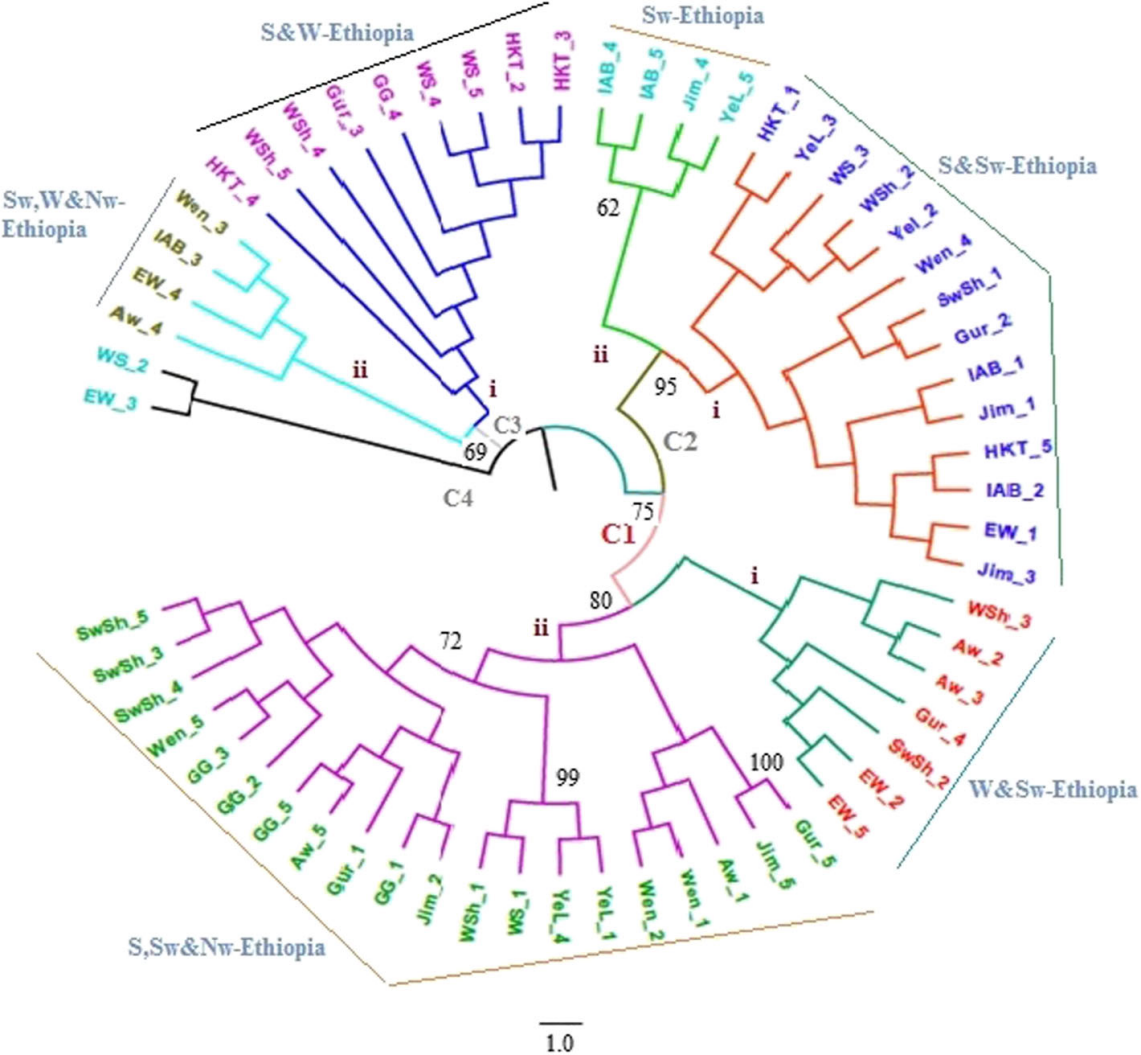
Neighbor-joining tree generated based on simple matching dissimilarity coefficients over 1000 replicates for 60 individual samples randomly selected from the 12 populations studied. Numbers at the roots of the branches are bootstrap values, and bootstrap values of less than 59% were not shown

**Fig. 3:**
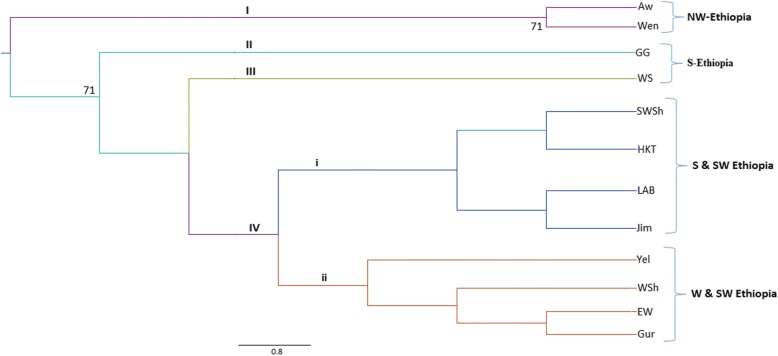
Unweighted pair-group method with arithmetic mean (UPGMA) dendrogram showing genetic relationships among the 12 populations considered based on Nei’s unbiased genetic distance over 1000 replicates. Numbers at the roots of the branches are bootstrap values, and bootstrap values of less than 59% were not shown

The neighbor-joining based cluster analysis of 60 individual samples randomly selected across the 12 populations (five samples per population) resulted in four major clusters (C1, C2, C3, and C4) with the first three clusters further divided into two sub-clusters. Each of the four clusters comprised individual plants from different collection zones (geographic regions). However, samples are more or less grouped according to their geographic region of origin at sub cluster levels (designated as i and ii on each major cluster) although there is considerable intermixes (Fig. 2). UPGMA [36] cluster analysis with bootstrap tests [39] has been conducted using Nei’s standard genetic distance at population level. The 12 populations formed four major clusters (I, II, III and IV) with cluster IV comprising eight populations that formed two sub-clusters (i and ii) (Fig. 3).

**Fig. 4:**
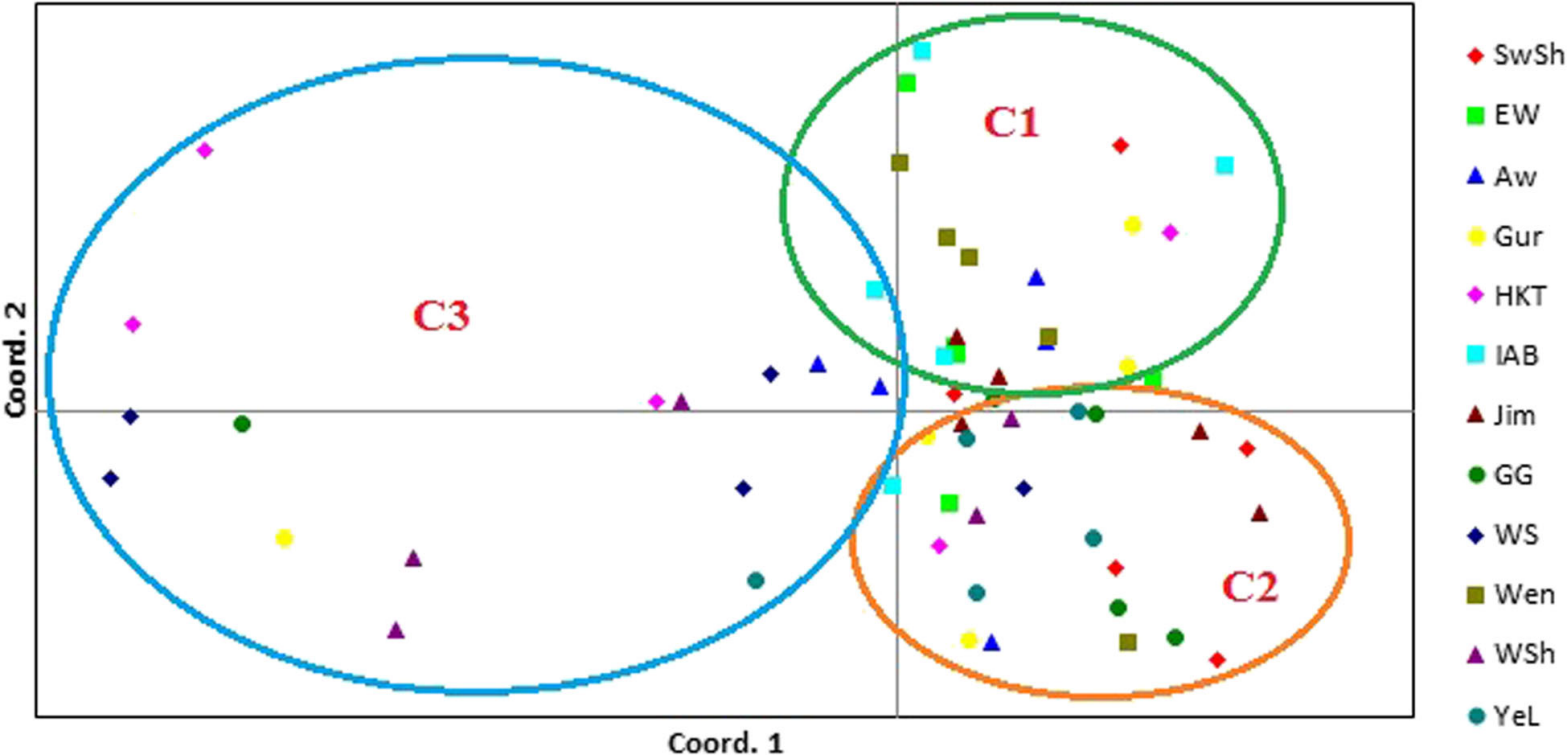
Principal coordinates analysis (PCoA) bi-plot showing the clustering pattern of 60 samples randomly selected from the 12 populations. Samples coded with the same symbol and color belong to the same population. Note: The percentages of variation explained by the first 3 axes (1, 2 and 3) are 14.1, 9.6, and 8.9%, respectively

A PCoA analysis revealed that the majority of samples were placed at the center of a two-dimensional coordinate plane (Fig. 4) forming roughly three groups (C1, C2 and C3), showing poor population structure. The first three axes explained 32% of the total variation.

**Fig. 5:**
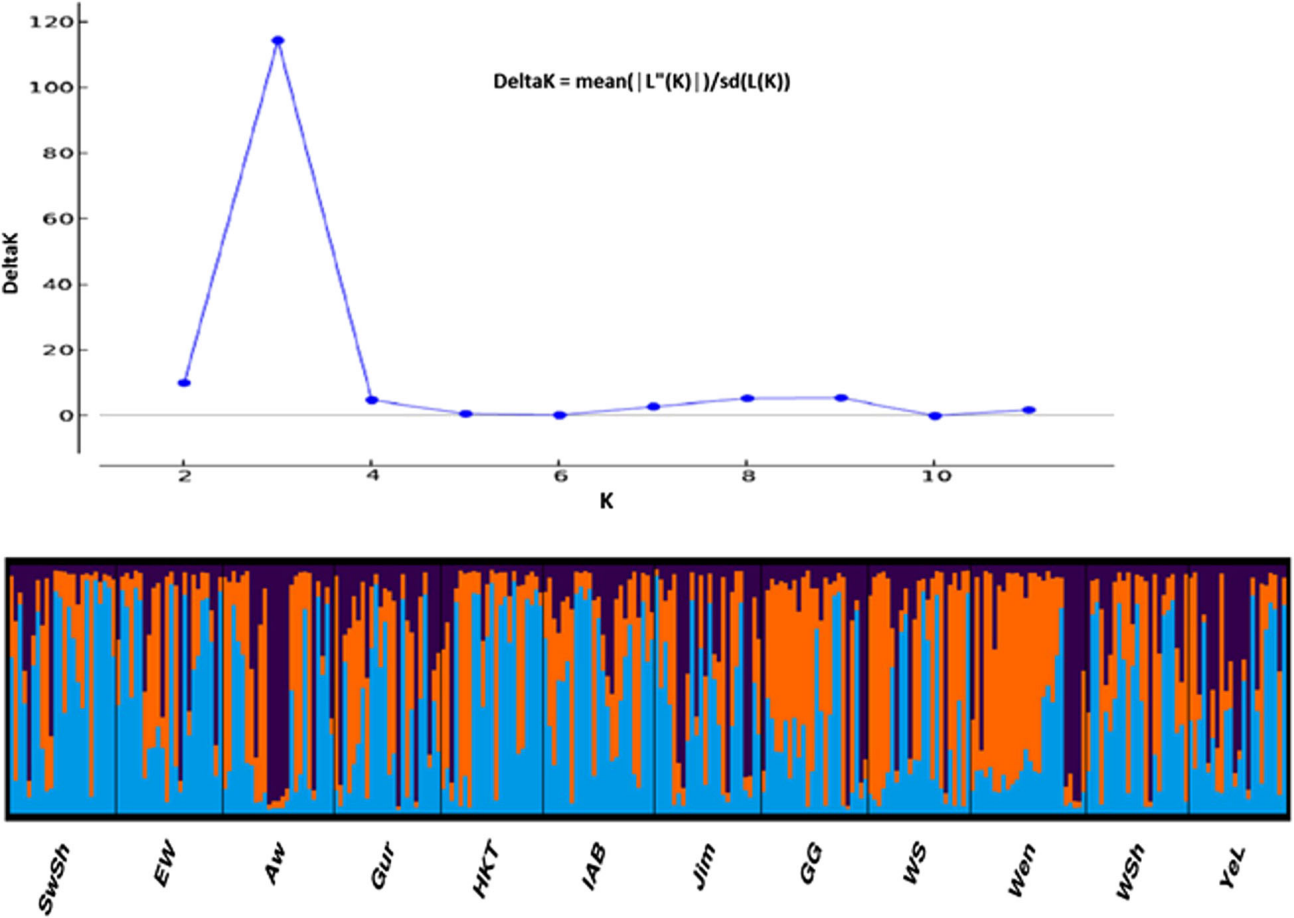
Delta K value estimated using Evano et al [44] method (**a**) and Bayesian model-based estimation of population structure (K = 3) (**b**) for the 287 *P. edulis* individual plants in twelve pre-determined populations

The Bayesian approach-based assignment of the 287 individual plants to different populations and determination of their population structure [45], using STRUCTURE outputs, predicted K = 3 to be the most likely number of clusters (Fig. 5a). Based on this value, Clumpak result (bar plot) showed wide admixtures and hence there was no clear geographic origin-based structuring of populations (Fig. 5b).

## Discussions

### EST-SSR markers validation and polymorphism evaluation

*P. edulis* is an indigenous tuber crop of Ethiopia that plays an indispensable role in food security of subsistence farmers in areas where it is cultivated. As the first step in the development of genomic tools and resources that can promote the conservation and breeding of this crop, we developed and validated 20 polymorphic EST-SSR markers. This work reports the transferability of SSR markers from *P. barbatus* to *P. edulis* and hence it enriches the limited reports on cross-genera [49] and cross-species [50] transferability of molecular markers, and molecular marker based genetic diversity analyses of Lamiaceae species. In the present study, the screening of 3263 *P. barbatus* ESTs resulted in 301 sequences (9.2%) containing SSRs. This proportion suggest that EST-SSRs are less abundant in *P. barbatus* than in *Salvia miltiorrhiza* (14.7%) [51] and *Lavandula* species (18.8%) [49], and slightly more abundant in *P. barbatus* than in *Mentha piperita* (8.4%) [52], which are all Lamiaceae species. The results also suggest that the abundance of the EST-SSRs in *P. barbatus* is higher than their abundance in cereal crops such as rice (4.7%), sorghum (3.6%), barley (3.4%) and maize (1.4%) [53]. However, other factors such as the SSR search criteria and size of the dataset might have partly contributed to the differences [54].

A maximum of two alleles are expected per individual plant at single copy microsatellite loci in diploid species. In the present study, a maximum of two alleles per plant were detected at each of the 20 loci, indicating that they are all single copy and have disomic inheritance regardless of the thus far reported basic chromosome number and ploidy level for several members of the genus *Plectranthus* [55]. However, the chromosome number of *P. edulis* has not been reported and, hence, further in-depth cytogenetic analysis is important to confirm the finding of this study.

The EST-SSR markers developed in the present study contained di-, tri- penta- and hexanucleotide repeats. Studies have shown that di, tri and tetra-nucleotide repeat SSRs are the most commonly used motifs in molecular genetic studies [56]. Tri-nucleotide repeat motifs were relatively abundant. This is attributed to the fact that they are more frequent in the EST’s coding regions, unlike in non-coding regions, in almost all taxa studied [54, 57–59] because of the positive selection for specific amino-acid stretches [60] or the prevalent selection against frameshift mutations in these regions for dinucleotides and other non-triplet repeat motifs [61]. As length and total size of perfect array of microsatellites increases, the frequency of repeats decreases and hence the informativeness increases [59, 62] owing to the higher mutation rates in longer microsatellites [63]. In agreement with this, the average number of repeats in dinucleotide SSRs is higher (8.7) than trinucleotides SSRs (5.7) among the SSRs developed in the present study. Similarly, the informativeness in terms of a number of alleles, GD and PIC was higher for di-nucleotide repeats than trinucleotides suggesting higher rate of evolution for SSRs with shorter repeat motifs than SSRs with longer repeat motifs.

The use of molecular markers for efficient selection of genotypes with desirable traits and enhancing the efficiency of breeding by allowing effective simultaneous selection of various desirable traits is a well-established approach [64, 65]. Hence, the large number of alleles detected in the present study suggests the suitability of microsatellites in general and those developed in this study in particular for genetic linkage and QTL mapping of desirable traits followed by marker assisted selection (MAS) in breeding programmes. However, most of the alleles were rare and scarce suggesting minimum selection pressure against the alleles. Otherwise, clonally propagating crops are expected to bear less proportion of such alleles as compared to seed-propagating crops. Moreover, the higher number of private alleles observed at several loci (Example: PE_07, PE_16, PE_18, PE_08) could offer a good opportunity to evaluate *P. edulis* genetic materials for the association of particular alleles with traits of interest and for conservation. Such alleles are useful in comparing diversity between species or populations [66] and also for measuring genetic distinctiveness of individuals in a population [67].

The average percent polymorphism per population revealed in the present study across the 20 loci (94%) is by far greater than the level reported by Kumar et al. [52] for 13 accessions of *M. piperita* (61%), which shares the same family with *P. edulis*. However, it was similar with that reported for 28 alfalfa accessions (97%) [68] and 37 *Opium poppy* accessions (96%) [69]. Such high percent polymorphism together with the PIC values obtained, which provides an estimate of the discriminatory power of a locus [70], and the allelic diversity suggest great potential of the markers for use in future genetic studies. However, the informativeness of a considerable number of loci is low and hence, there is a need to develop more highly informative EST-SSRs or other type of DNA markers that are suitable to characterize *P. edulis* genetic resources for efficient conservation and breeding.

The loci studied displayed differences between Ho and He in which half of them showed excess heterozygosity that led to a significant departure from HWE across populations. Such excess heterozygosity is expected in historically outcrossing species that maintain their heterozygosity through vegetative propagation, or if other factors such as natural and artificial selection pressure favor heterozygosity. Similar results have been reported in sweet potato [71, 72].

### Population genetic diversity

Higher genetic diversity is expected in larger and older populations when compared with small and newly established ones because of higher levels of accumulated and maintained genetic variation [73] which is important in increasing fitness and therefore reduces the likelihood of local extinction [74]. However, the mean observed heterozygosity (0.39), Shannon’s information index (0.61) and Nei’s gene diversity (0.35) obtained in the present study showed a medium level of genetic variation within populations. This could be mainly due to a relatively narrow genetic basis of the populations that resulted from limited germplasm resources accessible to farmers, or due to reduction in population size both due to natural as well as human factors, such as replacing cultivation of *P. edulis* by other crops. In addition, farmers’ preferences for selected traits of economic importance such as tuber size, tuber skin color, tuber texture, maturity etc. and asexual mode of reproduction of the crop (clonal propagation), which was evidenced from the considerably higher global clonality index (G/N), could have contributed to limited genetic variation in *P. edulis* populations. There are similar reports on potato cultivars from Yunnan province, China [75].

*Wen*, *WS*, and *Aw* populations are genetically more diverse than the other populations as estimated by parameters such as gene diversity, heterozygosity and Shannon diversity index and hence the areas representing these populations could be considered as genetic diversity hot spots and a potential in-situ conservation sites for *P. edulis*. Among the populations, *EW* has the least genetic diversity, which might suggest current rapid genetic erosion from the area (population bottleneck) or intensive artificial selection pressure to maximize tuber yield. In terms of allelic richness, *Gur*, *Jim*, *WS* and *WSh* populations are the top four in that order, and hence are more interesting in terms of genetic and evolutionary studies on this crop because allelic richness is more informative in this regard as it is sensitive to the presence of rare alleles [76] (which is prominent in this study) and population bottlenecks when compared to other parameters such as expected heterozygosity. Moreover, these populations except *WS* bear a relatively high proportion of private alleles which may indicate certain level of independent evolution of their gene pools that allowed maintenance of private alleles at a population level [77].

### Population genetic differentiation

AMOVA revealed that *P. edulis* has very low genetic differentiation among populations, which accounted only for 3% of the total genetic variation. The result is in line with previous reports on clonally propagating crops, such as *Ensete* (*Ensete ventricosum*) [78, 79], as such species tend to be more diverse within populations (but largely lower than sexually reproducing species) than among populations. Likewise, F_ST_ averaged across all loci (F_ST_ = 0.03) and pairwise F_ST_ for all pairs of populations (highest value = 0.07) was generally low to moderate on the bases of Wright [80] and Hartl and Clark [81] suggestions. Wright [80] indicated that genetic differentiation among populations can be considered high if the value of F_ST_ is greater than 0.25. This could be partly attained if gene flow, which is a powerful force to decrease differentiation among populations, is low (Nm < 1) [82]. Hence, the present study showed that *P. edulis* has very little population sub-structuring. The low population differentiation is supported by high gene flow (mean Nm = 18.29) owing to step-wise pollen movement across populations, germplasm exchange in the form of tubers and seeds through sharing common markets among several of the adjacent areas where different populations were collected. This study also showed the minimal effects of regions or geographic origins of populations on genetic variation in *P. edulis*. This could be partly explained by the extensive exchange of tubers as planting materials among farmers (gene flow), common origin of the populations, the clonally propagating nature of the crop in which only a limited number of individuals contribute tubers to the next generation, which gradually leads to recent or old population bottlenecks and hence facilitate genetic drift.

A pair-wise population genetic differentiation analysis resulted in a seven-fold variation in F_ST_ value among pairs of populations (ranging from 0.01 to 0.07). The highest population differentiation was observed between *Wen* and *HKT*, *Wen* and *WS*, *Wen* and *YeL* as well as between *Aw* and *WS* populations. *Wen* population showed the highest (0.05) pairwise Nei’s standard genetic distance with *WS* and *HKT* populations and is the most genetically distinct population with a mean Nei’s standard genetic distance 0.04 (Table 6). This can be partly explained by the fact that *Wen* and *Aw* populations were collected from a relatively pocket location and are separated from the other populations with a relatively longer geographic distance that probably restricted recent seed and tuber exchange. Hence, these populations may serve as potential sources of new genetic variation of important traits that can be used in breeding programs.

### Population genetic relationship and structure analysis

Neighbour joining cluster analysis in which each population is represented by five individual plants revealed a weak clustering pattern confirming low genetic differentiation among the populations and suggesting that the genetic background of *P. edulis* populations does not always correlate with their geographical origin. Although UPGMA and PCoA analyses also showed a certain level of populations clustering according to their geographical regions, the clustering pattern is weak to support the concept of “isolation by distance” [80]. Similarly, structure analysis revealed a close relationship (weak sub-division) among the samples from the 12 collection zones, and in general, three inferred groups (K = 3) with potential admixtures and have been observed. It is interesting to indicate that all individual plants analyzed have alleles originated from the three clusters, which supports the presence of a strong gene flow that led to poor population differentiation.

## Conclusions

In this study, we developed 20 EST-SSR markers for *P. edulis* based on EST sequences of *P. barbatus* deposited in the GenBank. All the markers were polymorphic in the populations studied and are valuable genetic tools to help evaluate the extent of genetic diversity and population structure of not only *P. edulis* but also of various species in the family Lamiaceae. These markers detected a larger number of alleles, some of which were private alleles that may be linked to important agronomic traits. The study also showed the potential of EST-SSR markers in defining how *P. edulis* genetic diversity is structured, and hence contribute to the development of better in-situ and ex-situ management strategies as well as selection criteria for the germplasm to be used in breeding programs for the improvement of various desirable traits in this crop. Among the 12 administrative zones and woredas considered, Wenbera, Awi and Wolaita have populations with a relatively high genetic diversity, and hence can be considered as hot spots for in-situ conservation of *P. edulis* as well as sources of desirable alleles for breeding values. Further studies that include germplasm from the remaining administrative zones and combine molecular characterization with agro-morphological analysis would be important to reveal additional potential sites for conservation and development of best-performing varieties. Overall, this study offered baseline information that promote further studies to exploit the high economic and endogenous values and to stop and reverse the current rapid genetic erosion of *P. edulis*.

## Additional files

Additional file 1: Passport data of the 174 tuber samples and 287 leaf samples representing the 12 populations used in the present study. (DOCX 36 kb)

Additional file 2: Allele frequency distribution and overall percentage of rare alleles (f ≤ 0.01) across populations. (XLSX 9 kb)

Additional file 3: Estimates of the overall Nei’s heterozygosity, population differentiation measures and proportion of progenies produced by selfing. (DOCX 18 kb)

## Abbreviations

AMOVA: Analysis of molecular variance;
CTAB: Cetyltrimethyl ammonium bromide
EST: Expressed sequence tags
NCBI: National Centre for Biotechnology information
PCoA: Principal coordinates analysis
SNNPs: Southern Nations, Nationalities, and Peoples’ Region
SSRs: Simple sequence repeats
UPGMA: Unweighted pair group with arithmetic mean
