# Supplementary material for: Genetic diversity and population structure analyses of Plectranthus edulis (Vatke) Agnew collections from diverse agro-ecologies in Ethiopia using newly developed EST-SSRs marker system
Source: BMC Genet. 2018 Oct 11;19:92. doi: 10.1186/s12863-018-0682-z (PMC6182789; doi:10.1186/s12863-018-0682-z)
Supplement: Supplementary file 1 — Passport data of the 174 tuber samples and 287 leaf samples representing the 12 populations used in the present study. (DOCX 36 kb) [file 12863_2018_682_MOESM1_ESM.docx]

Supplementary file 1: Passport data of the 174 tuber samples and 287 leaf samples representing the 12 populations used in the present study

| **Population name and size*** | **Coll. Code** | **Kebele** | **Altitude** | **Standard UTM** | | **Population name and size*** | **Coll. Code** | **locality** | **Altitude** | **Standard UTM** | |
| --- | --- | --- | --- | --- | --- | --- | --- | --- | --- | --- | --- |
|  |  |  |  | **Lat.** | **Long.** |  |  |  |  | **Lat.** | **Long.** |
| SwSh: 13 tuber and 24 leaf samples | ED001a, b | Gambela Goro | 1835m | 374792 | 927865 |  | ED088a,b | Yembero | 1879m | 219020 | 893136 |
|  | ED002a,b | Dambal Dildila | 1828m | 375795 | 930030 |  | ED089a,b | Yembero | 2096m | 219193 | 900059 |
|  | ED003a,b | Dambal Dildila | 1823m | 376028 | 930442 | Jim: 16 tuber and 24 leaf samples | ED090a | Balto | 1605m | 234630 | 870448 |
|  | ED004a,b | Dambi Kono | 1817m | 376129 | 930166 |  | ED091a | Bore | 1621m | 235207 | 871151 |
|  | ED005a,b | Dambi Kono | 1825m | 376350 | 930570 |  | ED092a | Gure Ganji | 1972m | 215625 | 861689 |
|  | ED006a,b | Kite Wato | 1915m | 381653 | 934072 |  | ED093a | Nasu | 1848m | 240990 | 875481 |
|  | ED007a,b | Kite Wato | 2599m | 378554 | 961153 |  | ED094a,b | Sadi | 1975m | 197673 | 855091 |
|  | ED008a,b | Kite Wato | 2612m | 378282 | 960973 |  | ED095a,b | Andode Mari | 1979m | 197291 | 855900 |
|  | ED009a,b | Leman Abo | 2631m | 373275 | 962106 |  | ED096a,b | Gibe Bosu | 1806m | 245128 | 841259 |
|  | ED010a | Foduna Gora | 1972m | 386105 | 940381 |  | ED097a,b | Keta Kedida | 2161m | 253103 | 844760 |
|  | ED011a,b | Foduna Gora | 1991m | 386714 | 940594 |  | ED098a,b | Keta Kedida | 2228m | 267135 | 829838 |
|  | ED012a | Foduna Gora | 2012m | 386594 | 940930 |  | ED099a,b | Buyo Kechema | 2181m | 267348 | 830162 |
|  | ED013a,b | Foduna Gora | 2003m | 386256 | 941347 |  | ED100a,b | Kofe | 1835m | 253193 | 844702 |
| EW: 14 tuber and 24 leaf samples | ED014a,b | Arele Waja | 1991m | 238222 | 1093812 |  | ED101a,b | Kofe | 1741m | 26224 | 846733 |
|  | ED015a | Arele Waja | 2060m | 236791 | 1094660 |  | ED102a | Yero Sokru | 1719m | 262234 | 846752 |
|  | ED016a,b | Arele Waja | 2124m | 236064 | 1095399 |  | ED103a | Wengesho | 1987m | 327451 | 874811 |
|  | ED017a | Sirba Wadessa | 2126m | 236158 | 1097468 |  | ED104a | Wengesho | 2010m | 327491 | 874631 |
|  | EDO18a,b | Kersa Albukane | 2087m | 239841 | 1094692 |  | ED105a | Yero Sokru | 1927m | 327078 | 875685 |
|  | ED019a,b | Warso | 2156m | 222300 | 1089267 | GG: 13 tuber and 24 leaf samples | ED106a,b | Boyina Tupa | 2655m | 343464 | 691930 |
|  | ED020a | Warso | 2166m | 222863 | 1098386 |  | ED107a | Mafuna Zola | 2666m | 343368 | 692737 |
|  | ED021a,b | Warso | 2149m | 221339 | 1089443 |  | ED108a | Mafuna Zola | 2678m | 343158 | 692308 |
|  | ED022a,b | Gode | 2151m | 221199 | 1089499 |  | ED109a,b | Gendo Gembela | 2705m | 342768 | 692333 |
|  | ED023a | Gode | 2142m | 221689 | 1089526 |  | ED110a | Losha | 2696m | 341154 | 690582 |
|  | EDO24a,b | Babo | 2172m | 266764 | 1103908 |  | ED111a | Losha | 2744m | 341322 | 690946 |
|  | ED025a,b | Babo | 2163m | 266545 | 1104268 |  | ED112a,b | Doko Shaye | 2733m | 340431 | 691145 |
|  | ED026a,b | Nole | 2141m | 266478 | 1105086 |  | ED113a,b | Gessa | 2522m | 333111 | 697778 |
|  | ED027a,b | Nole | 2151m | 266130 | 1104296 |  | ED114a,b | Gessa | 2746m | 333765 | 697306 |
| Aw: 21 tuber and 25 leaf samples | ED028a,b | Bida Jogla | 2554m | 271766 | 1209820 |  | ED115a,b | Ache | 2625m | 333653 | 696840 |
|  | ED029a | Bida Jogla | 2555m | 271844 | 1209745 |  | ED116a,b | Ache | 2602m | 332711 | 696733 |
|  | ED030a | Basa Enguana | 2610m | 275782 | 1213661 |  | ED117a,b,c | Egra | 2623m | 332363 | 696507 |
|  | ED031a | Basa Enguana | 2643m | 275760 | 1213723 |  | ED118a,b,c | Dorze | 2513m | 332638 | 697599 |
|  | ED032a | Kebele 02 | 2580m | 272444 | 1210394 | WS: 18 tuber and 23 leaf samples | ED119a,b | Sibaye Korke | 1979m | 372876 | 770184 |
|  | ED033a | Kebele 02 | 2541m | 272580 | 1211074 |  | ED120a | Sibaye Korke | 2012m | 372090 | 769633 |
|  | ED034a | Birntu Georgis | 2167m | 267632 | 1239283 |  | ED121a | Wendara Gale | 2027m | 370174 | 768940 |
|  | ED035a,b | Birntu Georgis | 2175m | 267210 | 1239531 |  | ED122a | Wandera Gale | 2034m | 370457 | 768351 |
|  | ED036a | Gafara | 2555m | 269860 | 1223160 |  | ED123a | Wandera Buluso | 2059m | 369715 | 763470 |
|  | ED037a | Gafara | 2590m | 269618 | 1221656 |  | ED124a,b | Shasha Gale | 2159m | 369409 | 764688 |
|  | ED038a | Sinisa | 2344m | 269504 | 1201176 |  | ED125a,b | Kokate Mare Chare | 2215m | 366537 | 760650 |
|  | ED039a,b | Ateta | 2380m | 269983 | 1202769 |  | ED126a | Delbo Atwaro | 2188m | 369768 | 762925 |
|  | ED040a,b | Ateta | 2385m | 269889 | 1202998 |  | ED127a | Delbo Atwaro | 2140m | 371214 | 762931 |
|  | ED041a | Birta | 2402m | 269625 | 1203898 |  | ED128a | Delbo Wegene | 2189m | 369255 | 762293 |
|  | ED042a | Birta | 2427m | 269710 | 1204516 |  | ED129a | Delbo Wegene | 2221m | 368812 | 762302 |
|  | ED043a | Sinisa | 2373m | 270095 | 1201499 |  | ED130a | Kokate Mare Chare | 2204m | 367925 | 761477 |
|  | ED044a | Jubaita Michael | 2460m | 281614 | 1201344 |  | ED131a | Kokate Mare Chare | 2232m | 367138 | 761189 |
|  | ED045a | Jubaita Michael | 2461m | 281419 | 1201272 |  | ED132a | Bolola Chew Kare | 2086m | 350087 | 764579 |
|  | ED046a | Jubaita Michael | 2488m | 281030 | 1202594 |  | ED133a | Bolola Chew Kare | 2099m | 349721 | 763493 |
|  | ED047a | Agza | 2486m | 281621 | 1203576 |  | ED134a,b | Doge Meshido | 2094m | 349417 | 763944 |
|  | ED048a | Agza | 2500m | 280702 | 1203809 |  | ED135a | Doge Meshido | 2109m | 349724 | 764974 |
| Gur: 18 tuber and 24 leaf samples | ED049a,b | Shawura | 2261m | 371729 | 867217 |  | ED136a,b | Doge Meshido | 2092m | 349195 | 764333 |
|  | ED050a | Wolicho | 2445m | 373024 | 867647 | Wen: 10 tuber and 26 leaf samples | ED137a,b,c | Babo | 2503m | 793823 | 1174693 |
|  | ED051a | Wolicho | 2253m | 372006 | 867995 |  | ED138a,b,c | Babo | 2517m | 793350 | 1174332 |
|  | ED052a,b | Jeda | 2382m | 373924 | 868624 |  | ED139a,b,c | Heto Shimo | 2513m | 793061 | 1174586 |
|  | ED053a | Jeda | 2379m | 374010 | 868572 |  | ED140a,b | Heto Shimo | 2530m | 792685 | 1174041 |
|  | ED054a | Jeda | 2350m | 371908 | 868375 |  | ED141a,b | Heto Shimo | 2476m | 792191 | 1174686 |
|  | ED055a,b | Gesabdi | 2126m | 372560 | 886833 |  | ED142a,b | Mana Sibu | 2481m | 790497 | 1174919 |
|  | ED056a | Gesabdi | 2131m | 372968 | 886340 |  | ED143a,b,c | Mana Sibu | 2504m | 789924 | 1175011 |
|  | ED057a | Amogera | 2155m | 373160 | 885715 |  | ED144a,b,c | Mana Sibu | 2483m | 789551 | 1174520 |
|  | ED058a | Amogera | 2176m | 374031 | 885462 |  | ED145a,b,c | Mana Sibu | 2509m | 790030 | 1175300 |
|  | ED059a | Okochira | 2069m | 371621 | 889342 |  | ED146a,b | Hambifata | 2501m | 789433 | 1174471 |
|  | ED060a | Kasaye | 2099m | 373406 | 889929 | WSh: 17 tuber and 23 leaf samples | ED147a | Ale Hula Dabi | 2924m | 324020 | 1007305 |
|  | ED061a | Kasaye | 2087m | 373025 | 889992 |  | ED148a | Ale Hula Dabi | 2898m | 322544 | 1004624 |
|  | ED062a | Isenina Dangeso | 2863m | 395550 | 879979 |  | ED149a,b | Ale Hula Dabi | 2918m | 322850 | 1005236 |
|  | ED063a | Isenina Dangeso | 2878m | 396749 | 880412 |  | ED150a,b | Toke Kombolcha | 2333m | 339287 | 990498 |
|  | ED064a | Bordana Damber | 2929m | 397159 | 880130 |  | ED151a,b | Toke Kombolcha | 2304m | 338799 | 989176 |
|  | ED065a | Bordana Damber | 2906m | 397588 | 879765 |  | ED152a,b | Toke Kombolcha | 2249m | 340335 | 990271 |
|  | ED066a,b,c | Harekit | 2895m | 397269 | 879266 |  | ED153a | Toke Irensa | 2252m | 340402 | 990196 |
| HKT: 9 tuber and 23 leaf samples | ED067a,b | Jawe | 2121m | 365839 | 830439 |  | ED154a | Liben Gamo | 2286m | 340673 | 990343 |
|  | ED068a,b | Jawe | 2161m | 367119 | 830378 |  | ED155a | Liben Gamo | 2249m | 340132 | 991042 |
|  | ED069a,b,c | Jawe | 2178m | 368204 | 830733 |  | ED156a | Golole Bolo | 2469m | 390882 | 991969 |
|  | ED070a,b,c | Sesh Duna | 2195m | 370720 | 832265 |  | ED157a,b | Golole Bolo | 2467m | 391246 | 992051 |
|  | ED071a,b,c | Sesh Duna | 2192m | 370744 | 832296 |  | ED158a,b | Sumbela Shiko | 2441m | 391789 | 992633 |
|  | ED072a,b | Wagebeta Eba | 2293m | 363184 | 813164 |  | ED159a | Shumbela Shiko | 2446m | 392269 | 992869 |
|  | ED073a,b,c | Wagebeta Eba | 2456m | 364593 | 813637 |  | ED160a | Shumbela Shiko | 2445m | 392667 | 992596 |
|  | ED074a,b | Ancho Sadicho | 2562m | 366420 | 813142 |  | ED161a | Gatira Lafto | 2449m | 393235 | 992986 |
|  | ED075a,b,c | Ancho Sadicho | 2556m | 366447 | 813134 |  | ED162a | Gatira Lafto | 2456m | 394228 | 993156 |
| IAB: 14 tuber and 25 leaf samples | ED076a,b | Yubo Mari | 1794m | 781347 | 900009 |  | ED163a | Gatira Lafto | 2460m | 394336 | 992132 |
|  | ED077a,b | Metu | 1851m | 781044 | 901224 | YeL: 11 tuber and 22 leaf samples | ED164a,b | Oya Keruwa | 2589m | 339188 | 873819 |
|  | ED078a | Metu | 1694m | 784997 | 916658 |  | ED165a,b | Oya Keruwa | 2540m | 339439 | 873081 |
|  | ED079a | Metu | 1696m | 784937 | 916755 |  | ED166a,b | Oya Keruwa | 2547m | 339275 | 873365 |
|  | ED080a | Wale | 1714m | 784744 | 916731 |  | ED167a,b | Oya Kepo | 2474m | 339716 | 875351 |
|  | ED081a,b | Tulema | 1650m | 775026 | 943622 |  | ED168a,b | Oya Kepo | 2470M | 339720 | 875350 |
|  | ED082a,b | Guji | 1664m | 775453 | 949107 |  | ED169a,b | Oya Kepo | 2466m | 339722 | 875348 |
|  | ED083a,b | Humbe | 1923m | 183276 | 927973 |  | ED170a,b | Oya Ereto | 2560m | 338923 | 874054 |
|  | ED084a,b | Digaja | 1919m | 133324 | 927001 |  | ED171a,b | Oya Ereto | 2570m | 338941 | 874058 |
|  | ED085a,b | Urgessa | 1947m | 183233 | 928938 |  | ED172a,b | Oya Ereto | 2558m | 338927 | 874051 |
|  | ED086a,b | Sobo | 1901m | 210424 | 938681 |  | ED173a,b | Deri | 2479m | 337472 | 875661 |
|  | ED087a,b | Sida | 1915m | 240425 | 938683 |  | ED174a,b | Deri | 2474m | 337470 | 875666 |

**SwSh = Southwest Shewa; EW = Esat Wollega; Aw = Awi; Gur = Gurage; HKT = Hadiya, Kembata-Tembaro; IAB = Illu Aba Bora; Jim = Jimma; GG = Gamo Gofa; WS = Wollaita Sodo; Wen = Wenbera; WSh = West Shewa; YeL = Yem Liyu ; ‘ED’ is the code for ‘Ethiopian Dinich’; letters: a, b, c next to numbers represent the individual plants used for leaf sample collection per tuber sample; number of tuber and leaf samples of each population are given after colon;; Latitude and Longitude readings are in standard UTM*
