# Supplementary material for: Genetic diversity and population structure analyses of Plectranthus edulis (Vatke) Agnew collections from diverse agro-ecologies in Ethiopia using newly developed EST-SSRs marker system
Source: BMC Genet. 2018 Oct 11;19:92. doi: 10.1186/s12863-018-0682-z (PMC6182789; doi:10.1186/s12863-018-0682-z)
Supplement: Supplementary file 3 — Estimates of the overall Nei’s heterozygosity, population differentiation measures and proportion of progenies produced by selfing. (DOCX 18 kb) [file 12863_2018_682_MOESM3_ESM.docx]

**Supplementary file** **3:**

Estimates of the overall Nei’s heterozygosity, population differentiation measures and proportion of progenies produced by selfing

| Locus | Hs | Fis | Gis | DST | Fst | Gst | Fit | S | HW* |
| --- | --- | --- | --- | --- | --- | --- | --- | --- | --- |
| PE_01 | 0.232 | 0.057 | 0.071 | 0.014 | 0.063* | 0.058 | 0.115*** | 0.121 | 0.008** |
| PE_02 | 0.553 | -0.756 | -0.758 | 0.001 | 0.003 | 0.002 | -0.752 | -0.861 | 0.999ns |
| PE_03 | 0.239 | 0.215*** | 0.219 | 0.005 | 0.023*** | 0.020 | 0.233 | 0.548 | 0.000*** |
| PE_04 | 0.158 | -0.127 | -0.115 | 0.006 | 0.050 | 0.039 | -0.074** | -0.225 | 0.894ns |
| PE_05 | 0.357 | 0.258*** | 0.246 | 0.007 | 0.020*** | 0.019 | 0.273 | 0.695 | 0.000*** |
| PE_06 | 0.694 | -0.241 | -0.247 | 0.009 | 0.016 | 0.013 | -0.221 | -0.388 | 0.999ns |
| PE_07 | 0.052 | 0.751*** | 0.723 | 0.000 | 0.011*** | 0.008 | 0.755 | 6.032 | 0.000*** |
| PE_08 | 0.079 | 0.136* | 0.162 | 0.002 | 0.033** | 0.029 | 0.164* | 0.315 | 0.003** |
| PE_09 | 0.500 | 0.243*** | 0.237 | 0.004 | 0.009*** | 0.007 | 0.251 | 0.642 | 0.000*** |
| PE_10 | 0.404 | -0.331 | -0.333 | 0.014 | 0.034 | 0.032 | -0.285*** | -0.497 | 0.999ns |
| PE_11 | 0.380 | -0.025 | -0.026 | 0.005 | 0.015 | 0.012 | -0.009 | -0.049 | 0.551ns |
| PE_12 | 0.386 | 0.245*** | 0.249 | 0.008 | 0.024*** | 0.021 | 0.264 | 0.649 | 0.000*** |
| PE_13 | 0.078 | 0.536*** | -0.026 | -0.001 | 0.011*** | -0.010 | 0.530 | 2.310 | 0.000*** |
| PE_14 | 0.286 | -0.045 | -0.037 | 0.021 | 0.074 | 0.068 | 0.030*** | -0.086 | 0.241ns |
| PE_15 | 0.183 | 0.290*** | 0.272 | 0.005 | 0.032*** | 0.028 | 0.313* | 0.817 | 0.000*** |
| PE_16 | 0.441 | -0.115 | -0.104 | 0.021 | 0.049*** | 0.046 | -0.062*** | -0.206 | 0.89ns |
| PE_17 | 0.488 | -0.149 | -0.167 | 0.023 | 0.052** | 0.045 | -0.086*** | -0.259 | 0.979ns |
| PE_18 | 0.488 | 0.101* | 0.109 | 0.002 | 0.007*** | 0.005 | 0.108 | 0.225 | 0.003** |
| PE_19 | 0.531 | -0.658 | -0.654 | 0.002 | 0.004 | 0.003 | -0.652 | -0.794 | 0.999ns |
| PE_20 | 0.388 | -0.401 | -0.395 | 0.019 | 0.051 | 0.046 | -0.330*** | -0.572 | 0.999ns |
| Overall | 0.346 | -0.122 | -0.127 | 0.008 | 0.026*** | 0.024 | -0.093 | 0.421 | 0.999ns |

Hs = Gene diversity within populations; DST = Nei’s (1978) unbiased average gene diversity between subpopulations; Fis = Inbreeding coefficient within individuals; Gis = Inbreeding coefficient within individuals, adjusted for bias; Fst = Inbreeding coefficient within subpopulations relative to total (genetic differentiation among subpopulations); Gst = Analog of Fst, adjusted for bias or Wright (1951) Fst; Fit = Inbreeding coefficient of an individual relative to the total population (Total genetic differentiation); S = 2*FIS/ (1-FIS); HW* = Overall Hardy-Weinberg test using Fit; * p<0.05, ** p<0.01, ***p<0.001
